# Evaluation of the genetic diversity of a set of parents of *Eucalyptus spp*. by using microsatellite markers to direct matings

**DOI:** 10.1186/1753-6561-5-S7-P55

**Published:** 2011-09-13

**Authors:** Lucas Santos, Eullaysa Sabóia, Douglas Almeida, Jupiter Abad, Alexandre Missiaggia, Norma Pereira, Dário Ahnert, Fernanda Gaiotto, Ronan Corrêa

**Affiliations:** 1 Universidade Estadual de Santa Cruz, Brazil; 2 Universidade Estadual do Sudoeste da Bahia, Brazil; 3 Centro de Tecnologia – Fibria Celulose S.A., Brazil

## Background

To obtain genetically superior cultivars in a breeding program, methods and procedures are necessary to allow the identification of selected individuals over several cycles of selection while at the same time maintain broad genetic base of the breeding populations. This is crucial to guarantee continuous genetic gains along the program. The establishment of efficient breeding strategies depends on methods and analytical tools. The assessment of genetic diversity with molecular markers of parents used in mating designs could aid optimizing the recombination phase. Microsatellites provide good information content and require small amounts of DNA and may be transferable between species of the same genus. In this study we evaluated the genetic diversity in a set of *Eucalyptus* parent trees and indicated those to be preferentially crossed in a recombination process to potentially maximize variation in the offspring for individual selection of clones.

## Materials and methods

A total of 20 genotypes were selected from 44 families obtained by crossing 24 parents in different types of hybridizations of populations of *Eucalyptus grandis* and *E.urophylla*. Genomic DNA was extracted from leaves and amplified with microsatellites developed during the Genolyptus project and previously used for *Eucalyptus spp*[1]. The amplifications were performed using the GeneAmp PCR System 9600 (Applied Biosystems) thermal cycler using the following amplification cycle: 96 ° C for 2 min, 30 cycles at 94 ° C for 1 min, specific primer annealing temperature for 1 min, 72 ° C for 1 min and a final extension step at 72 ° C for 7 min. Loci were amplified in single and duplex system. Tocher clustering were developed using the genetic distance matrix with the software Genes.

## Results and conclusions

This study showed wide genetic diversity among the 20 genotypes under evaluation; all nine SSR loci analyzed were polymorphic, with a total of 77 alleles. The number of alleles per locus ranged from 5 (EMBRA 646) to 10 (Embra 645) averaging 9.1. The lowest value of polymorphism was obtained for marker EMBRA 915 (PIC = 0.59) and higher for the marker EMBRA 645 (PIC = 0.89). The expected heterozygosity ranged from 0.66 to 0.89, averaging 0.80. Based on genetic distances, the 20 genotypes were clustered into six distinct groups. The greatest genetic distance was observed between genotypes C022H (GIV) and M4320G (GVI), and the smallest among genotypes P023H (GI) and C407 (GII). Group II clustered the largest number of genotypes (C386H, C407H, C053H, C051H, TC10H, P044H, VR3748H, VR3709, P4295H), consistent with the fact that these genotypes are considered to belong to the same "heterotic" group by the breeders. Based on the genetic distances, the following trees were selected to be preferentially crossed: M4320H, P4295H, P082G, VR3709H, C053H, C219H, C041H and C022H. It remains to be seen if the progenies derived form these crosses will in fact display a wider segregation and allow, as expected, effective selection of extreme phenotypes for growth and wood quality.
